# Circulating DNA addresses cancer monitoring in non small cell lung cancer patients for detection and capturing the dynamic changes of the disease

**DOI:** 10.1186/s40064-016-2141-5

**Published:** 2016-04-26

**Authors:** Zhangjing Wei, Nirej Shah, Chong Deng, Xuehui Xiao, Tenglang Zhong, Xiansong Li

**Affiliations:** Department of Diagnostic Medicine, JingMen NO.1 People’s Hospital, JingMen, People’s Republic of China; Department of Clinical Medicine, JingChu University of Technology, JingMen, People’s Republic of China; Department of Neurosurgery, JingZhou Hospital, Tongji Medical College, Huazhong University of Science and Technology (HUST), Renmin Road 1, JingZhou, 434020 People’s Republic of China

**Keywords:** Non small cell lung cancer (NSCLC), T790M mutation, cfDNA, ctDNA, Drug resistance

## Abstract

**Purpose:**

Monitoring of key markers for lung cancer detection and tracking of acquired drug resistance is critical for the management of the disease. We aim to ascertain the value of monitoring both total cell free DNA concentrations and mutant EGFR DNA content within diverse groups of individuals most vulnerable to the disease.

**Methods:**

We proposed longitudinal monitoring of circulating DNA using digital PCR. Circulating DNA present in peripheral blood can be obtained non-invasively and provide timely disease status update. 25 heavy smokers and 50 patients undergoing TKI therapy were recruited. Peripheral blood specimens were taken at different time points and their circulating DNA were analyzed and quantified.

**Results:**

Significant higher concentrations of total cell free DNA were detected when compared with healthy high-risk individuals. Levels were stable throughout the treatment cycles, which makes it potentially a useful tool for patient stratification. Concurrent mutant T790M DNA detection of lung cancer patients at baseline achieved 82 % concordance with matched tissue analysis. Samples initially negative for the T790M gene mutation that became positive during treatment were corroborated with a repeat biopsy. The results showed its usefulness for serial monitoring.

**Conclusion:**

Monitoring of circulating DNA addresses the need for disease detection and shows the ability to capture the dynamic changes of the disease.

**Electronic supplementary material:**

The online version of this article (doi:10.1186/s40064-016-2141-5) contains supplementary material, which is available to authorized users.

## Background

Non small cell lung cancer (NSCLC) is the leading cause of cancer death and tyrosine kinase inhibitors (TKIs) have emerged as crucial treatment options (Lynch et al. 2004). However, most patients inevitably show disease progression due to several different mechanisms (Sequist et al. 2011). The most common is a single secondary mutation in exon 20 of the EGFR gene (T790M; Ohashi et al. 2013). As a result of the bulkier methionine residue at position 790, it abrogates the inhibitory role of the TKI (Pao et al. 2005). The median time to disease progression is approximately 12 months (Kobayashi et al. 2005). Acquired resistance remains the lethal weakness for the use of TKIs and it is meaningful to monitor the emergence of T790M mutation.

In this regard, repeat tissue biopsies can provide critical information of patients’ current disease status but physical constraints such as ill health and reluctance for surgery do not allow for longitudinal monitoring. Tumor tissues are also heterogeneous (Bedard et al. 2013) which is challenging to accurately profile the disease. Liquid biopsy via circulating DNA in cancer patients is an attractive source for tumor analysis as it offers real time monitoring with a simple blood draw (Diaz and Bardelli 2014). The mutant DNA phenomenon has been extensively investigated for lung cancer in recent years (Watanabe et al. 2015; Thress et al. 2015). The DNA is typically fragmented and found in the cell free component of whole blood. It was initially reported by Mandel and Metais in 1948 (Mandel and Metais 1948) and is now found to be useful in many disciplines of medicine (Papageorgiou et al. 2011; Diehl et al. 2008). Isolating circulating DNA from individuals may yet be another effective means to detect and track cancer.

Here we aim to show individuals at higher risk of lung cancer and patients who are on therapy to benefit from detecting and tracking circulating DNA. Through concurrent monitoring of cfDNA in blood plasma and measuring the quantity of T790M EGFR mutation that confers TKI resistance in patients, we hope to ascertain a detection routine that will aid to identify high-risk patients. Our results showed that cfDNA is stable over time and significantly elevated for patients. This presents opportunities of utilizing cfDNA as a cancer biomarker. Furthermore, longitudinal studies of blood samples provided real time monitoring of changes to the disease mutational profile.

## Methods

### Study cohort and trial approvals

50 NSCLC patients undergoing treatment within our clinics were recruited for this study as approved by the Institutional Review Board (IRB). Patients were on EGFR TKI treatment and divided into two groups based on their EGFR T790M mutation status. Details are provided in Table 1. Tumor genotyping was performed during routine clinical care. Baseline blood samples were taken within a week after the first dose of TKI. A total of 25 patients were enlisted in each group and all patients consented to be part of the study. Selected patients whose results were discordant with the primary tumor analysis after 6 months of monitoring underwent a repeat biopsy for confirmation studies. Additionally, 25 healthy volunteers who are smokers but had been tested to be disease free were recruited as well. Healthy volunteers underwent a detailed clinical examination and/or radiographic imaging to confirm their disease free status.

**Table 1:** Patients suffering from NSCLC and on TKI treatment were divided into two groups based on T790M positivity

*Group 1 patients*	
Sample size	25
Mutational status	T790M negative (mixture of L858R and exon19 del)
Age	45–70
Gender	
Male	15
Female	10
Smoker status	
Yes	20
No	5
Disease stage	IV
Histology	Adenocarcinoma
*Group 2 patients*	
Sample size	25
Mutational status	T790M positive (mixture of L858R and exon19 del)
Age	56–68
Gender	
Male	16
Female	9
Smoker status	
Yes	18
No	7
Disease stage	IV
Histology	Adenocarcinoma
*Healthy volunteers*	
Sample size	25
Age	40-67
Gender	
Male	15
Female	10
Smoker status	
Yes	25
No	0

### Plasma sample processing

Peripheral blood was collected from each patient during follow-up visits. Blood samples were drawn in 10 ml EDTA vacutainers and processed within 2 h. The process was the same for healthy volunteer’s blood sampling. Plasma extraction was performed by centrifuging the blood samples at 1000*g* for 10 min at 4 **°**C and the supernatant carefully transferred into a separate collection tube. A repeat centrifugation step was performed on the supernatant to remove any remaining cells. cfDNA was extracted from these samples using Qiagen’s QIAamp Circulating Nucleic Acid kit (Qiagen Inc., USA) following manufacturer’s instructions. Approximately 5 ml of plasma were processed from each sample and stored at −20 **°**C prior to molecular analysis. Blood specimens from the healthy volunteers were also subjected to the same processing steps.

### Detection of mutant EGFR, T790M via ddPCR

Droplet digital PCR (ddPCR) was done on a QX100 ddPCR system (BioRad, USA). The assay provides the sensitivity to detect low levels of mutant target DNA in each sample. Primers and probes for T790M detection were adopted from Oxnard et al. (2014). cfDNA extracted from the blood samples were analyzed for T790M mutation at each sampling time point. Validation runs with control specimens were prepared using plasmids containing T790M and wild type EGFR.

### Measurement of cell free DNA quantity

Total cfDNA after extraction was quantified using Quant-iT™ PicoGreen^®^ dsDNA Assay Kit (ThermoFisher, USA) following manufacturer’s instructions. Briefly, the PicoGreen reagent was freshly prepared and each reaction contained 50 μl of working solution and sample DNA. Every sample was examined in duplicates. Measurements were taken on a fluorometer (Tecan, USA) at wavelengths of 520 nm (emission) and 480 nm (excitation). Standard curves were produced with Lambda DNA by serial dilution. The average values of two replicates were taken as the final measurement.

### Statistical analysis

In order to analyze the correlation between patients’ mutant DNA in blood and that of the tissue biopsies, the Cohen’s kappa was used to deduce the significance between the two variables. Comparisons of cfDNA in healthy volunteers’ and patients’ specimens were done using the Student’s t test. A receiver-operating curve (ROC) was plotted for healthy volunteers against patients with lung cancer to evaluate the suitability of cfDNA analysis as a detection assay. Survival analysis was performed with the Kaplan–Meier estimate and the overall survival (OS) of patients was compared using the log-rank test. All categorical variables were represented as mean ± standard deviation unless otherwise specified. All statistical analyses were calculated using the Prism software (GraphPad, San Diego, USA).

## Results

### Study design and detection assay characteristics

**Fig. 1:**
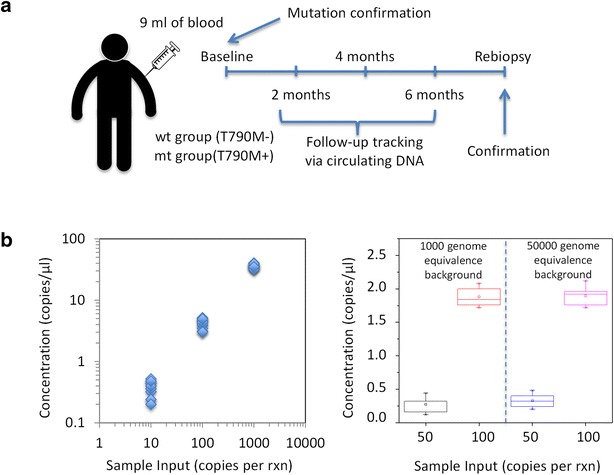
Clinical monitoring of circulating DNA in NSCLC patients. **a** Clinical specimen characteristics and follow up measurement schedules. **b** Assay sensitivity to detect minute quantities of mutant copies via ddPCR

Our study is a systematic analysis to investigate the potential utility of circulating DNA in three critical cohorts. We compared lung cancer patients receiving EGFR-TKI treatment (Fig. 1a) with high-risk healthy volunteers who are smokers with more than 20 pack-years. Patients who had advanced or recurrent NSCLC were divided into two main groups, one with the drug resistance mutation T790M and the other group lacking this mutation. Molecular profiling was done using tissue biopsies when the patients were first diagnosed. Peripheral blood samples were collected at specific time points during patient follow-ups. A second blood sampling of healthy volunteers was done after 6 months from the initial draw. All patients were on TKI treatment of either gefitinib or erlotinib for the duration of the study. Details of patient characteristics are provided in Table 1. During each sampling, both total cfDNA and copies of mutant DNA were measured. At the end of the study, patients with discordant data from their initial baseline results underwent a repeat biopsy for confirmation.

In order to track copies of T790M DNA, we utilized a ddPCR assay, which has the sensitivity to detect low numbers of target mutant DNA in a pool of wildtype background. This is critical as the prevalence of mutant DNA in total cell free plasma can be very low (Watanabe et al. 2015). We designed controlled experiments with spiked inputs per 25 μl reactions as shown in Fig. 1b to validate the assay. Every input condition was repeated ten times to ensure the specificity and sensitivity. Our validations demonstrated good linearity at differing amounts of target DNA inputs. Linear regression of the data points showed a r^2^ value of 0.98. The mean detected copies of mutant DNA was close to the spiked input amount demonstrating excellent sensitivity. In a background of 1000–50,000 wildtype copies, the assay successfully picked up the mutant DNA. Concentrations as low as 50 mutant to 50,000 wildtype copies were used and the results were consistent at different background conditions.

### Circulating DNA of study cohorts at baseline

**Fig. 2:**
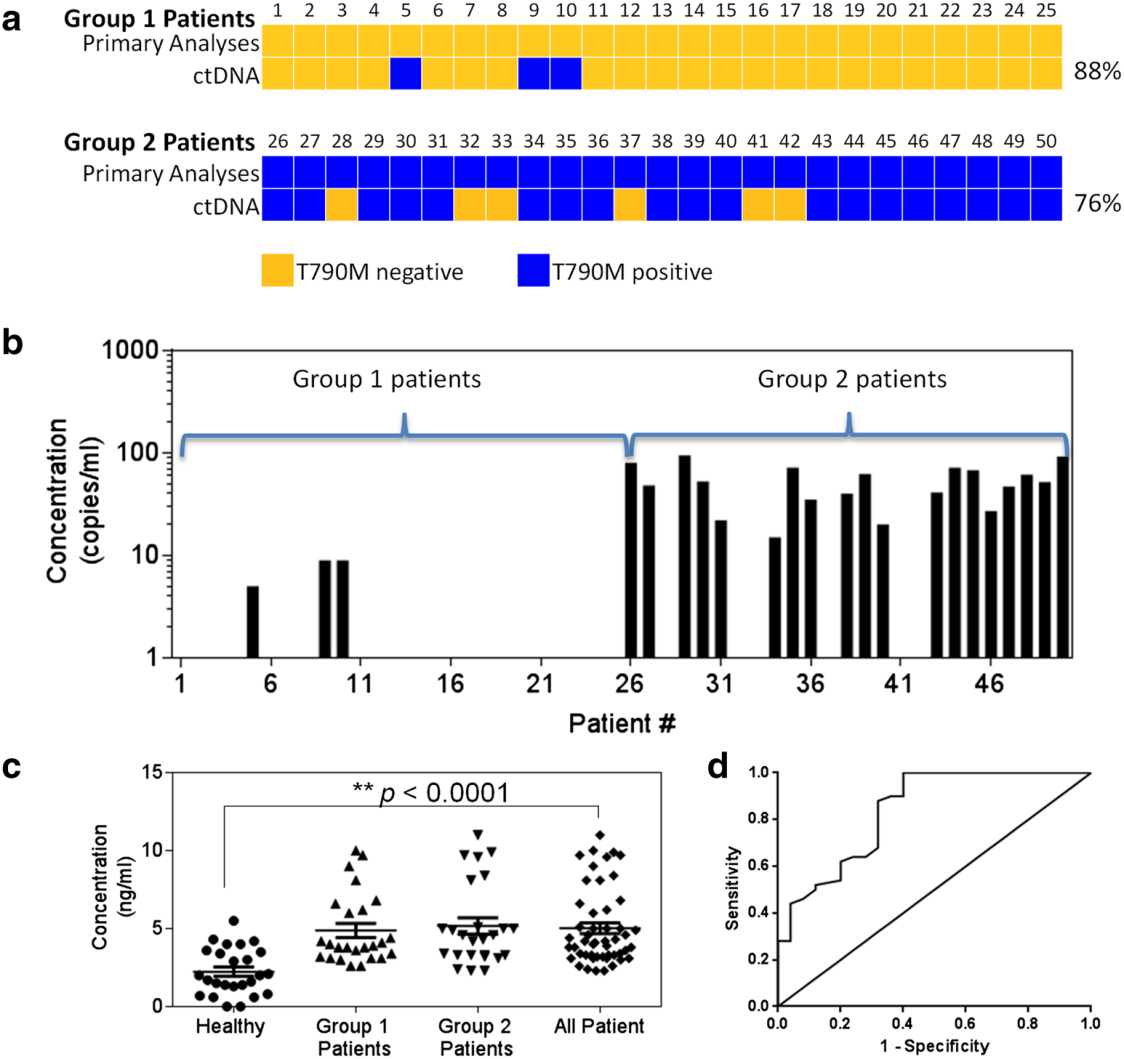
Profiles of NSCLC patients’ circulating DNA quantity taken at baseline. **a** Concordance measurement of mutant EGFR status using both tissue biopsies and ctDNA analysis. **b** Quantity of T790M mutant DNA across different patient samples. **c** cfDNA levels measured across different patients comparing with healthy volunteers. **d** ROC analysis for healthy volunteers against lung cancer patients

The plasma DNA for healthy volunteers and the two groups of patients were assessed at the start of the study to determine the initial concordance rate. For group 1 patients that had negative T790M status from tissue analyses, three (patients #5, #9 and #10) were found to harbor the resistance mutation from blood sampling. Samples were analyzed in replicates and both tests were positive. Overall the concordance rate for group 1 was 88 % as shown in Fig. 2a. Group 2 patients with T790M positive profiles were found to have six discordant cases (patients #28, #32, #33, #37, #41, #42). 76 % of the samples from ctDNA were established to be in agreement with the primary tissue analysis. Collectively, 82 % of all samples had concordant genotypes when compared to their respective primary biopsies’ results (Cohen’s kappa, κ = 0.64, Additional file 1: Figure S1A). All healthy volunteers were tested negative for the mutant gene (data not shown). Mutant DNA quantities assessed by ddPCR are compiled in Fig. 2b. The range of DNA concentrations vary between 5 and 95 copies/ml and mean detection was 46.6 ± 26.9 copies/ml for patients with positive T790M genotypes.

Concurrently, quantities of total cfDNA extracted from different study groups were compared. We found a statistical significance of higher total cfDNA for patient samples as compared to healthy volunteers with a *p* value <0.0001 as shown in Fig. 2c. However, it was statistically indeterminate between group 1 and group 2 patient cohorts. Average recovered cfDNA from healthy volunteers was 2.24 ± 1.50 ng/ml and patient specimens yielding 5.03 ± 2.44 ng/ml. For the three positive patients in group 1, total cell free DNA quantities were 9.0 (#5), 2.6 (#9) and 4.4 (#10) ng/ml. Except for patient #5 whose total cell free DNA concentration was above the 75th percentile of the group, patients #9 and #10 were closer to the 25th and 50th percentile respectively.

The area under the ROC for healthy volunteers against the 50 lung cancer patients was 0.84 (95 % CI 0.74–0.94) as shown in Fig. 2d. For healthy volunteers comparing with group 1 patients, the area was 0.83 (95 % CI 0.72–0.95) and comparing with group 2 patients, the area was 0.85 (95 % CI 0.7388–0.9524) (Additional file 1: Figures S1B and S1C).

### Longitudinal monitoring of circulating DNA

**Fig. 3:**
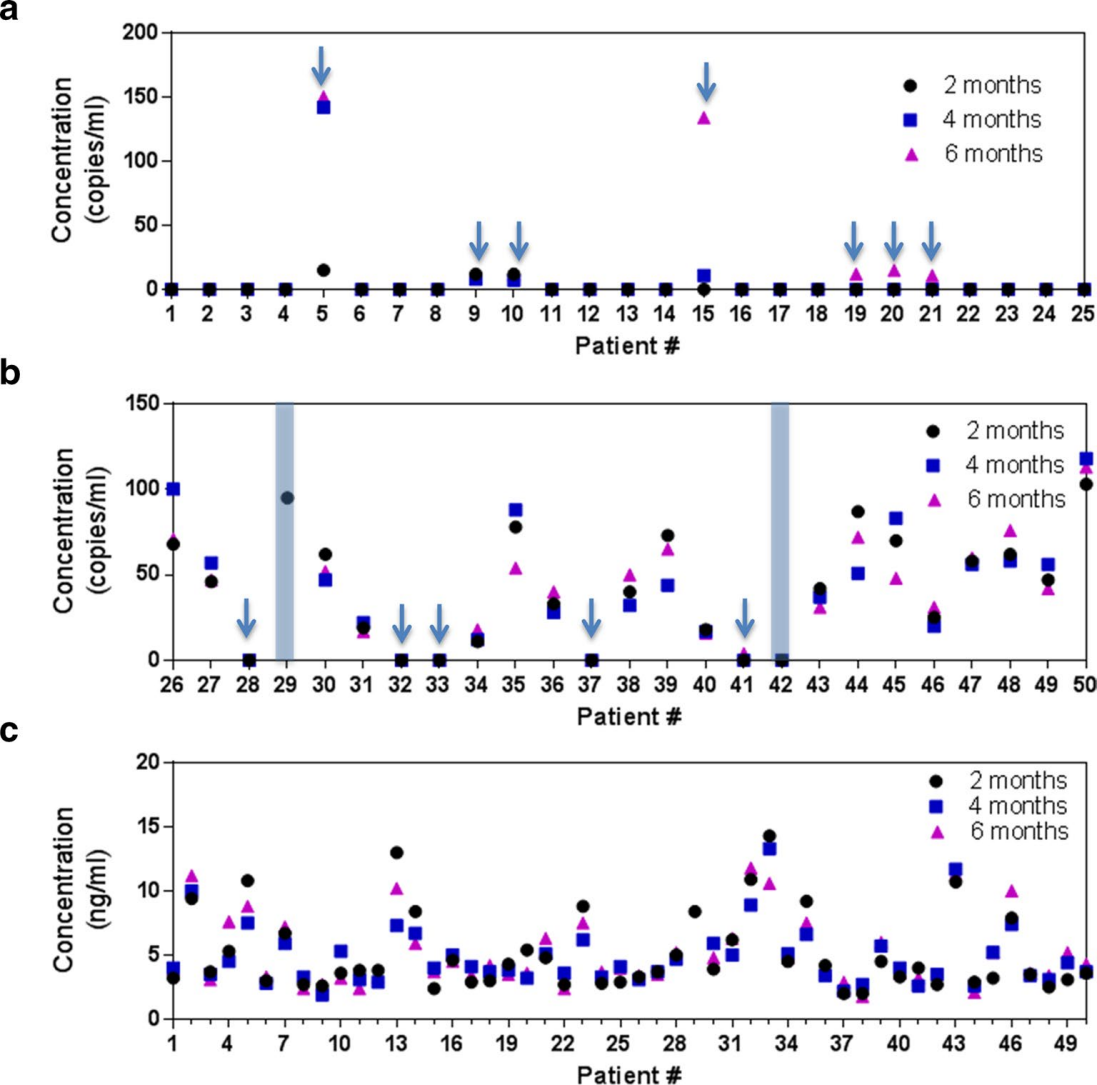
Serial monitoring of circulating DNA in the plasma during the treatment process. **a** Patient group that did not harbor T790M at baseline. 7 samples indicated the presence of T790M with ctDNA analysis at the end of 6 months. **b** Patient group that harbored T790M at baseline. Patients 29 and 32 died before the end of the study and specific data points cannot be obtained. 2 samples indicated the absence of T790M mutations in ctDNA. **c** cfDNA serial measurements for the 2 groups of patients for disease monitoring

In this current prospective study, we examined the circulating DNA of patients undergoing treatment and variations of healthy volunteers over an extended time period. In group 1, four additional patients were observed to have a positive mutant EGFR detection besides patients #5, #9 and #10 who were detected at baseline (Fig. 3a). Patient #15 was identified at the second follow-up roughly 4 months after the first blood test, while patients #19, #20 and #21 were detected after 6 months. Interestingly, we also observed patient #5 having a sharp increase in mutant DNA concentration after 4 months while concentrations for patients #9 and #10 remained constant throughout. All other specimens were not detected for the mutant DNA during the entire serial monitoring period.

For group 2 (Fig. 3b), two participants died before the completion of the trial and serial data points were lacking (highlighted by the regions in the blue boxes). Of the initial six patients that had discordant results, only patient #41 was positively identified after 6 months. Excluding the two datasets that were incomplete, nine patients showed an overall decrease in mutant DNA after 6 months while five remained relatively unchanged when compared to their respective baseline measurements. Unlike group 1 patients, we did not observe any cases with large variations of detected mutant EGFR copies. Similar observations were also seen in total cfDNA concentrations as noted in Fig. 3c. Specimen #13 from group 1 showed the largest change with a two-fold reduction comparing the results at 2 and 4 months. Variations in the entire study cohort ranges from 0.2 to 5.7 ng/ml. Total cfDNA quantities within healthy volunteers (Additional file 1: Figure S2) were stable as well. We were able to obtain blood specimens from 23 of 25 patients with an average concentration of 1.77 ng/ml, a marginal decline from 2.24 ng/ml at baseline. The average change in concentration for the measured 23 volunteers was 0.59 ng/ml.

In addition, OS was assessed in group 1 and 2 patients to compare their survival outcomes. As shown in Additional file 1: Figure S3, patients who initially had mutant EGFR copies had a worse outcome. Hazard ratio was determined to be 3.25 with *p* value <0.001 using a log rank test.

### Repeat biopsies confirm ctDNA derived genotypes

**Fig. 4:**
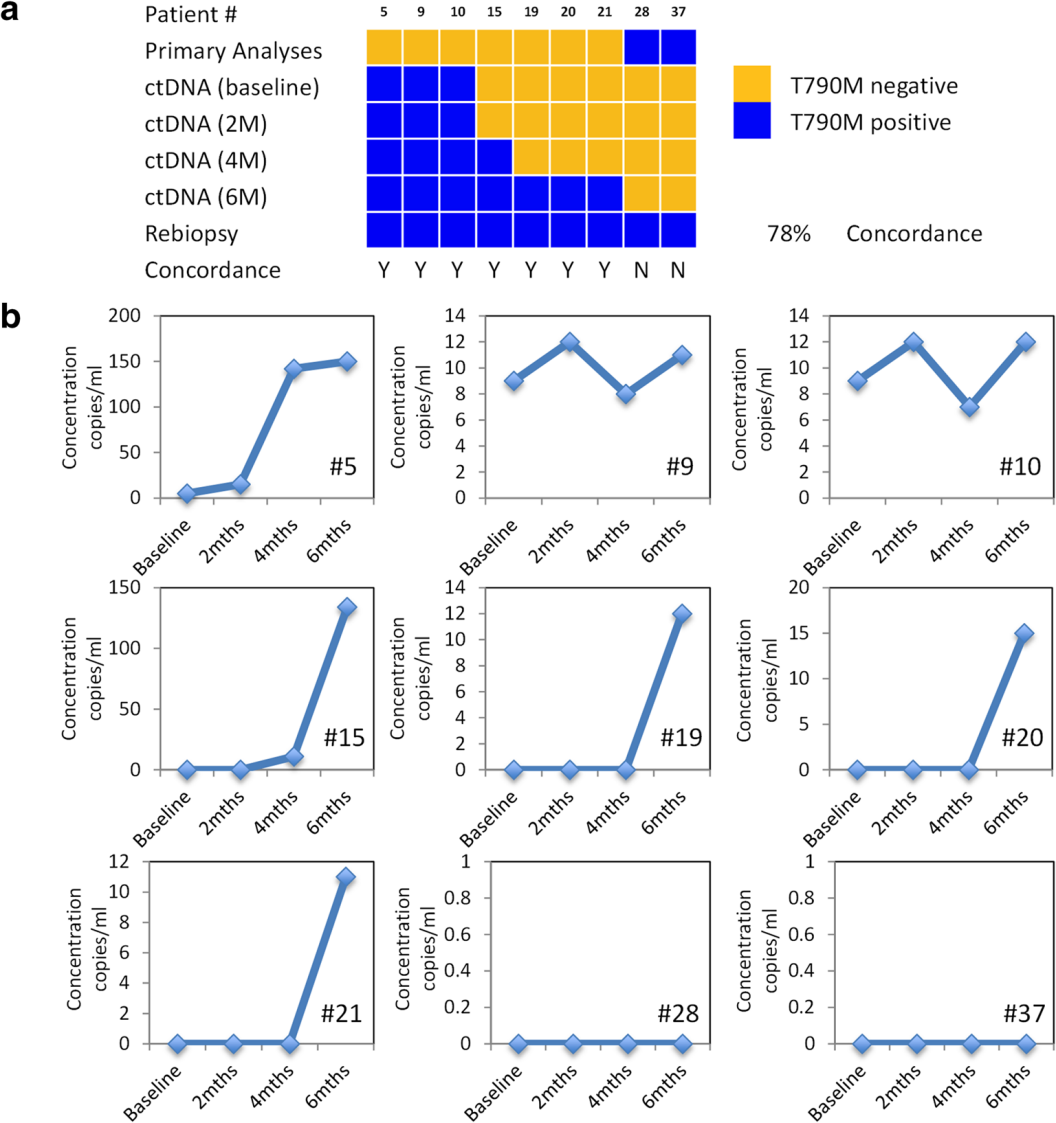
Concordance measurement of selected samples with repeat biopsies performed. **a** Summary of different patient profiles. **b** Detailed measurements of mutant DNA concentrations during monitoring

Patients identified during the monitoring phase with discordant mutant EGFR results with their initial tissue diagnosis underwent a repeat biopsy as part of the study. The results are highlighted in Fig. 4. Patients #5, #9 and #10, which were detected during baseline measurements using blood plasma were positive in the following repeat biopsy. Patients #15, #19, #20 and #21 that were subsequently identified during the course of monitoring were positive as well. Overall all group 1 patients who were identified during the monitoring period were concordant with the final repeat biopsy. However, discrepancies were seen in patients #28 and #37 from group 2. Initial and repeat biopsies were positive for the resistance mutation but negative in all mutant DNA samples.

## Discussion

Our study was designed to determine the utility of circulating DNA for clinical oncology especially for early detection and monitoring of lung cancer. Prior studies had focused much attention on patients in different stages of the disease (Douillard et al. 2014; Kim et al. 2013; Xie et al. 2004; Leon et al. 1977), and our work has further extended to various critical groups. We designed the investigation to target high-risk individuals and patients resistant to EGFR-TKI therapy, which is one of the most prevalent complications for managing the disease. The incidence of EGFR mutations is estimated at around 77 % among NSCLC clinical responders to gefitinib or erlotinib (Sharma et al. 2007) and the T790M mutation alone accounts for 50 % of all cases. It is thus important that patients are closely monitored for aberrations to the gene. Our studies uncovered several interesting findings. Notably, total cfDNA is useful at baseline examination to identify potential cancer patients. In addition, the changes in mutant DNA status can be a clear indication of secondary EGFR mutations evolving in tumors. Consequently, we strongly believe both total cfDNA quantities and its mutational signatures may both be exploited for diagnostics and monitoring purposes.

Our measurements of cfDNA in healthy volunteers as well as NSCLC patients showed several trends. Variations of cfDNA quantity were small across different groups throughout the monitoring period. Furthermore, NSCLC patients were shown to have consistently higher absolute amounts above healthy volunteers. This suggest the levels of total cfDNA is stable at different time points and the significantly elevated amounts associated with NSCLC patients advocates its use for early detection of the disease. A recent study (Szpechcinski et al. 2015) was determined to be 90 % sensitive and 80.5 % specific for cancer patients comparing with a normal person’s plasma, which concurs with our results. We further showed that heavy smokers who are of greater risk to lung cancer have two fold lesser concentration of cfDNA than patients undergoing TKI treatment. ROC analysis showed good accuracy with an area larger than 0.8 for employing such a test. In addition, we observed that total cfDNA quantity did not change significantly with increase in mutant DNA amounts as shown by patients #5 and #15. We also did not observe any significant trends within the two study groups although most patients in group 1 responded well to treatment. Our results are consistent with several other studies that measured the effects of total cfDNA concentrations. Gautschi et al. (2004) and Tissot et al. (2015) both measured its variation post chemotherapy and discovered no association to therapy. Taken together, the stability of cfDNA concentration as measured in our longitudinal study and the significantly lower amounts detected in healthy volunteers showed that cfDNA can be a potentially useful parameter for early detection of lung cancer.

With the concurrent mutant EGFR DNA detection, we yielded good concordance with the primary tissue analysis. This is coherent with published literature (Watanabe et al. 2015; Sundaresan et al. 2015; Oxnard et al. 2014) where efforts have been made to find suitable non-invasive methods for monitoring T790M mutations. Using similar ultra sensitive methods involving ddPCR, Masaru et al. identified the mutant gene in 79.9 % of samples (Watanabe et al. 2015), which is similar to our results at 76 % for group 2 patients. Additionally, we found it was useful to target samples that did not present the mutant gene from tissue biopsies. In our analysis of baseline measurements on group 1 cohort, we uncovered three patients with positive detection that were subsequently verified in the repeat biopsies as true positives. This is likely the result of intra-tumoral heterogeneity (Gerlinger et al. 2012) where the position of tissue sampling matters. Current liquid biopsy methods shown in this study can further complement existing guidelines to better gain an accurate diagnosis of the patient’s disease profile.

The main benefits of employing liquid biopsy to manage cancer are the ease and non-invasiveness of the technique while gaining real time status of how the disease is evolving. Traditional core needle biopsies are an invasive procedure that requires a skilled personal. Furthermore, there are possibilities that inadequate tissues are taken or conditions of the patients do not allow for a biopsy (Kim et al. 2011). In a study conducted by Sundaresan et al. (2015), 23 % of patients failed to recover sufficient material for genotyping. Our study will aid such cases and we have shown that this is helpful in a number of group 1 patients. Patients #15, #19, #20 and #21 were detected at various stages of their treatment regime, which were corroborated with the final repeat biopsies. Survival analysis of group 1 and 2 patients highlighted a worse outcome for patients with mutant copies. It is therefore important for constant monitoring to detect changes promptly. The early detection of the changes in the gene will allow for quicker clinical invention and permit better-tailored treatments as the disease evolves. The monitoring regime coupled with prompt clinical intervention, such as switching to 3rd generation TKIs, will hopefully impact clinical outcomes in the future.

In summary, our data suggest that clinical monitoring using circulating free DNA is beneficial for lung cancer patients both in terms of early detection and monitoring of drug response for acquired resistance. Blood sampling is a commonly performed procedure that is relatively non-invasive and can be used to actively monitor cancer patients. Our study demonstrated that detecting mutant DNA can aid to discover changes within the disease much faster and has immediate clinical utility. This work focused on various groups most vulnerable to the disease. More importantly, we were able to track its dynamic changes. Within this dataset of 75 individuals, we showed that concentrations of total cfDNA were relatively stable throughout the treatment process and remained elevated above high-risk healthy individuals. This strongly suggests the potential as a diagnostic tool and will have to be validated thoroughly with a larger cohort study. A future potential study will also include clinical invention upon detecting the mutant DNA to address clinical outcomes. The methods and procedures we have highlighted in this study will better elucidate the dynamic nature of cancer during the detection and treatment process.

## Additional file

10.1186/s40064-016-2141-5 Comparison of circulating DNA of different study groups at baseline. (A) Cohen’s kappa analysis. (B) ROC depicting the relation of healthy volunteers against group 1 patient. (C) ROC for evaluating the usefulness of cfDNA as a screening mechanism. **Figure S2**. cfDNA concentration of healthy volunteers at different time points. **Figure S3**. Overall survival of patients showing group 2 individuals having worse outcomes.

## References

[CR1] Bedard PL, Hansen AR, Ratain MJ, Siu LL (2013). Tumour heterogeneity in the clinic. Nature.

[CR2] Diaz LA, Bardelli A (2014). Liquid biopsies: genotyping circulating tumor DNA. J Clin Oncol.

[CR3] Diehl F, Schmidt K, Choti MA, Romans K, Goodman S, Li M, Thornton K, Agrawal N, Sokoll L, Szabo SA, Kinzler KW, Vogelstein B, Diaz LA (2008). Circulating mutant DNA to assess tumor dynamics. Nat Med.

[CR4] Douillard JY, Ostoros G, Cobo M, Ciuleanu T, Cole R, McWalter G, Walker J, Dearden S, Webster A, Milenkova T, McCormack R (2014). Gefitinib treatment in EGFR mutated caucasian NSCLC: circulating-free tumor DNA as a surrogate for determination of EGFR status. J Thorac Oncol.

[CR5] Gautschi O, Bigosch C, Huegli B, Jermann M, Marx A, Chasse E, Ratschiller D, Weder W, Joerger M, Betticher DC, Stahel RA, Ziegler A (2004). Circulating deoxyribonucleic acid as prognostic marker in non-small-cell lung cancer patients undergoing chemotherapy. J Clin Oncol.

[CR6] Gerlinger M, Rowan AJ, Horswell S, Larkin J, Endesfelder D, Gronroos E, Martinez P, Matthews N, Stewart A, Tarpey P, Varela I, Phillimore B, Begum S, McDonald NQ, Butler A, Jones D, Raine K, Latimer C, Santos CR, Nohadani M, Eklund AC, Spencer-Dene B, Clark G, Pickering L, Stamp G, Gore M, Szallasi Z, Downward J, Futreal PA, Swanton C (2012). Intratumor heterogeneity and branched evolution revealed by multiregion sequencing. N Engl J Med.

[CR7] Kim ES, Herbst RS, Wistuba II, Lee JJ, Blumenschein GR, Tsao A, Stewart DJ, Hicks ME, Erasmus J, Gupta S, Alden CM, Liu S, Tang X, Khuri FR, Tran HT, Johnson BE, Heymach JV, Mao L, Fossella F, Kies MS, Papadimitrakopoulou V, Davis SE, Lippman SM, Hong WK (2011). The BATTLE trial: personalizing therapy for lung cancer. Cancer Discov.

[CR8] Kim HR, Lee SY, Hyun DS, Lee MK, Lee HK, Choi CM, Yang SH, Kim YC, Lee YC, Kim SY, Jang SH, Lee JC, Lee KY (2013). Detection of EGFR mutations in circulating free DNA by PNA-mediated PCR clamping. J Exp Clin Cancer Res.

[CR9] Kobayashi S, Boggon TJ, Dayaram T, Janne PA, Kocher O, Meyerson M, Johnson BE, Eck MJ, Tenen DG, Halmos B (2005). EGFR mutation and resistance of non-small-cell lung cancer to gefitinib. N Engl J Med.

[CR10] Leon SA, Shapiro B, Sklaroff DM, Yaros MJ (1977). Free DNA in the serum of cancer patients and the effect of therapy. Cancer Res.

[CR11] Lynch TJ, Bell DW, Sordella R, Gurubhagavatula S, Okimoto RA, Brannigan BW, Harris PL, Haserlat SM, Supko JG, Haluska FG, Louis DN, Christiani DC, Settleman J, Haber DA (2004). Activating mutations in the epidermal growth factor receptor underlying responsiveness of non-small-cell lung cancer to gefitinib. N Engl J Med.

[CR12] Mandel P, Metais P (1948). Les acides nucleiques du plasma sanguin chez l’homme. C R Seances Soc Biol Fil.

[CR13] Ohashi K, Maruvka YE, Michor F, Pao W (2013). Epidermal growth factor receptor tyrosine kinase inhibitor-resistant disease. J Clin Oncol.

[CR14] Oxnard GR, Paweletz CP, Kuang Y, Mach SL, O’Connell A, Messineo MM, Luke JJ, Butaney M, Kirschmeier P, Jackman DM, Jänne PA (2014). Noninvasive detection of response and resistance in EGFR-mutant lung cancer using quantitative next-generation genotyping of cell-free plasma DNA. Clin Cancer Res.

[CR15] Pao W, Miller VA, Politi KA, Riely GJ, Somwar R, Zakowski MF, Kris MG, Varmus H (2005). Acquired resistance of lung adenocarcinomas to gefitinib or erlotinib is associated with a second mutation in the EGFR kinase domain. PLoS Med.

[CR16] Papageorgiou EA, Karagrigoriou A, Tsaliki E, Velissariou V, Carter NP, Patsalis PC (2011). Fetal-specific DNA methylation ratio permits noninvasive prenatal diagnosis of trisomy 21. Nat Med.

[CR17] Sequist L, Waltman B, Dias-Santagata D, Digumarthy S, Turke A, Fidias P, Bergethon K, Shaw A, Gettinger S, Cosper A, Akhavanfard S, Heist R, Temel J, Christensen J, Wain J, Lynch T, Vernovsky K, Mark E, Lanuti M, Iafrate A, Mino-Kenudson M, Engelman J (2011). Genotypic and histological evolution of lung cancers acquiring resistance to EGFR inhibitors. Sci Transl Med.

[CR18] Sharma SV, Bell DW, Settleman J, Haber DA (2007). Epidermal growth factor receptor mutations in lung cancer. Nat Rev Cancer.

[CR19] Sundaresan TK, Sequist LV, Heymach JV, Riely GJ, Janne PA, Koch WH, Sullivan JP, Fox DB, Maher R, Muzikansky A, Webb A, Tran HT, Giri U, Fleisher M, Yu H, Wei W, Johnson BE, Barber TA, Walsh JR, Engelman JA, Stott SL, Kapur R, Maheswaran S, Toner M, Haber DA (2015). Detection of T790M, the acquired resistance EGFR mutation, by tumor biopsy versus noninvasive blood-based analyses. Clin Cancer Res.

[CR20] Szpechcinski A, Chorostowska-Wynimko J, Struniawski R, Kupis W, Rudzinski P, Langfort R, Puscinska E, Bielen P, Sliwinski P, Orlowski T (2015). Cell-free DNA levels in plasma of patients with non-small-cell lung cancer and inflammatory lung disease. Br J Cancer.

[CR21] Thress KS, Brant R, Carr TH, Dearden S, Jenkins S, Brown H, Hammett T, Cantarini M, Barrett JC (2015). EGFR mutation detection in ctDNA from NSCLC patient plasma: a cross-platform comparison of leading technologies to support the clinical development of AZD9291. Lung Cancer.

[CR22] Tissot C, Toffart A-C, Villar S, Souquet P-J, Merle P, Moro-Sibilot D, Pérol M, Zavadil J, Brambilla C, Olivier M, Couraud S (2015). Circulating free DNA concentration is an independent prognostic biomarker in lung cancer. Eur Respir J.

[CR23] Watanabe M, Kawaguchi T, S-i Isa, Ando M, Tamiya A, Kubo A, Saka H, Takeo S, Adachi H, Tagawa T, Kakegawa S, Yamashita M, Kataoka K, Ichinose Y, Takeuchi Y, Sakamoto K, Matsumura A, Koh Y (2015). Ultra-sensitive detection of the pretreatment EGFR T790M mutation in non-small cell lung cancer patients with an EGFR-activating mutation using droplet digital PCR. Clin Cancer Res.

[CR24] Xie GS, Hou AR, Li LY, Gao YN, Cheng SJ (2004). Quantification of plasma DNA as a screening tool for lung cancer. Chin Med J.

