# Supplementary material for: Circulating DNA addresses cancer monitoring in non small cell lung cancer patients for detection and capturing the dynamic changes of the disease
Source: Springerplus. 2016 Apr 26;5:531. doi: 10.1186/s40064-016-2141-5 (PMC4844578; doi:10.1186/s40064-016-2141-5)
Supplement: Supplementary file 1 — 10.1186/s40064-016-2141-5 Comparison of circulating DNA of different study groups at baseline. (A) Cohen’s kappa analysis. (B) ROC depicting the relation of healthy volunteers against group 1 patient. (C) ROC for evaluating the usefulness of cfDNA as a screening mechanism. Figure S2. cfDNA concentration of healthy volunteers at different time points. Figure S3. Overall survival of patients showing group 2 individuals having worse outcomes. [file 40064_2016_2141_MOESM1_ESM.docx]

**Circulating DNA addresses cancer monitoring in non small cell lung cancer patients for detection and capturing the dynamic changes of the disease**

Zhangjing Wei^1^, Nirej Shah^2^, Chong Deng^1^, Xuehui Xiao^1^, Tenglang Zhong^1^ Xiansong Li^3*^


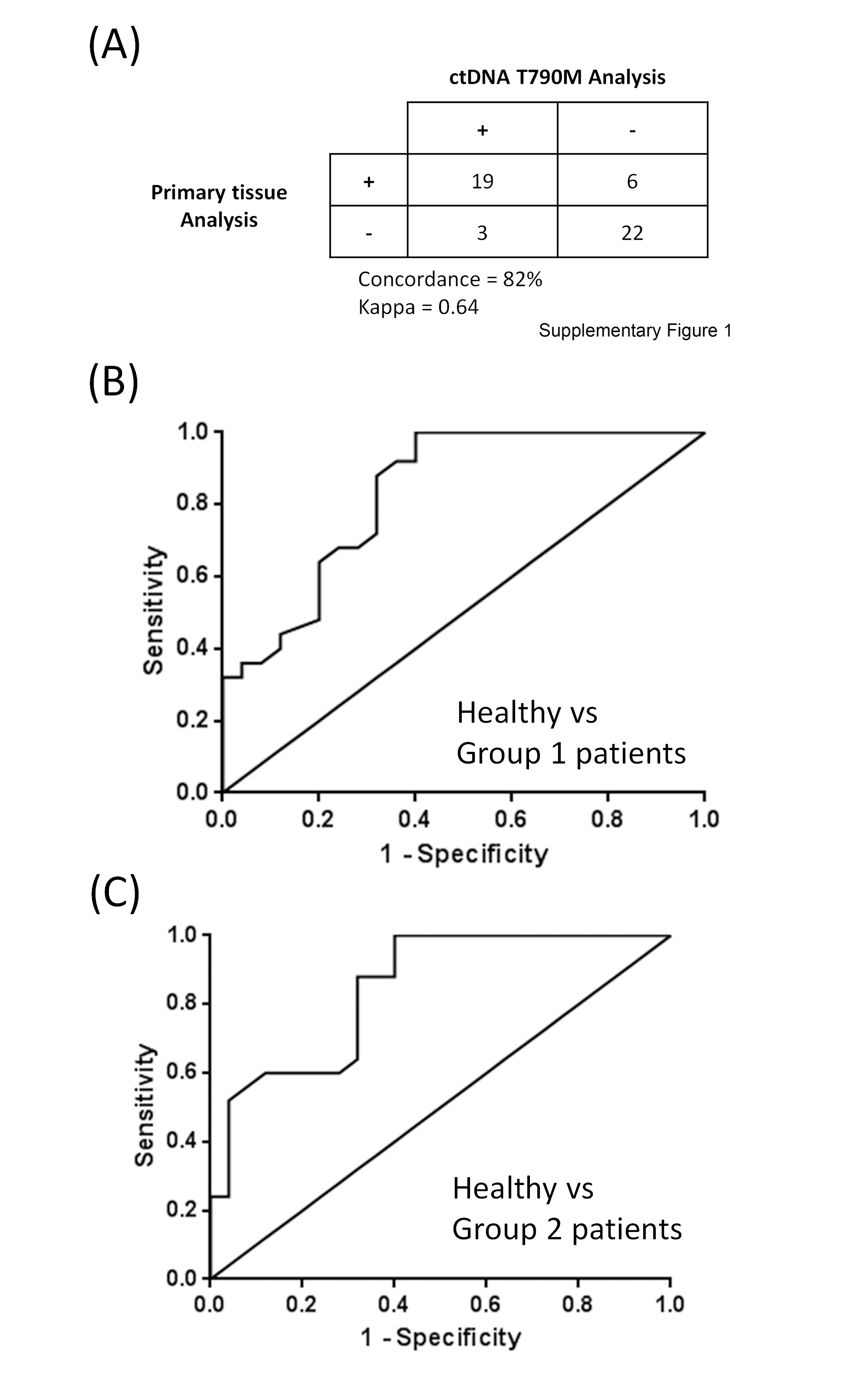


**Figure S1.** Comparison of circulating DNA of different study groups at baseline. (A) Cohen’s kappa analysis. (B) ROC depicting the relation of healthy volunteers against group 1 patient. (C) ROC for evaluating the usefulness of cfDNA as a screening mechanism.


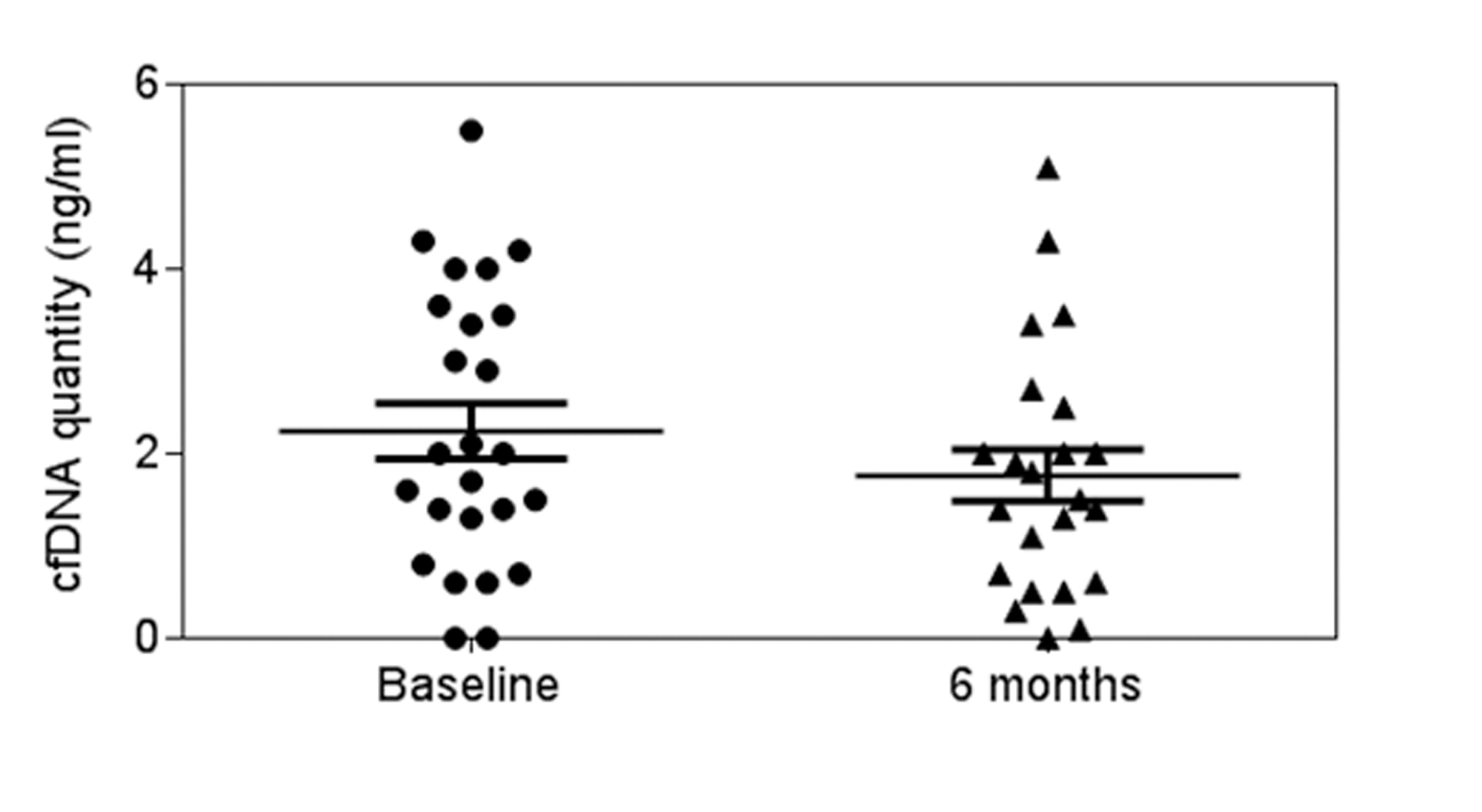


Figure S2. cfDNA concentration of healthy volunteers at different time points


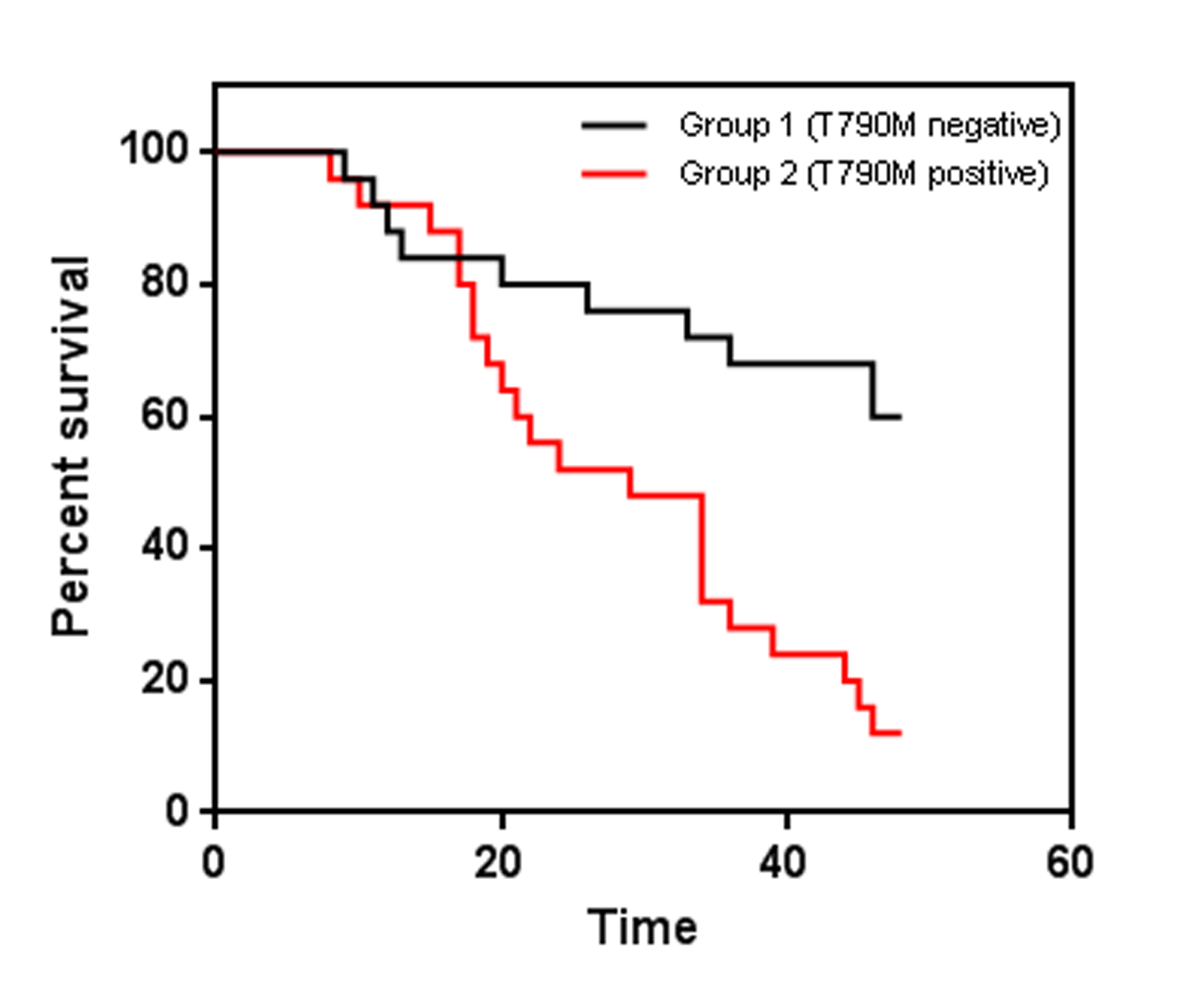


Figure S3. Overall survival of patients showing group 2 individuals having worse outcomes.
